# A Retrospective Chart Review of Uninsured Encounters in a Primary Care Hospital in Japan

**DOI:** 10.7759/cureus.110782

**Published:** 2026-06-13

**Authors:** Junki Mizumoto, Yumiko Hironaka

**Affiliations:** 1 Department of Family Practice, Ehime Seikyo Hospital, Matsuyama, JPN; 2 Center for General Medicine Education, School of Medicine, Keio University, Tokyo, JPN; 3 Regional Medical Coordination Office, Ehime Seikyo Hospital, Matsuyama, JPN

**Keywords:** healthcare equity, healthcare inequity, lack of health insurance, social determinants of health, unequal access to healthcare

## Abstract

Introduction

Lack of health insurance is known to delay care-seeking and contribute to preventable morbidity. Although Japan has established a universal health insurance system, more than 10% of households have unpaid insurance premiums, and approximately 2.5% possess insurance certificates with restricted or no validity. Under these circumstances, preventable deaths attributable to delayed medical care for financial reasons have been reported. Individuals facing unstable employment or job loss are particularly vulnerable to premium non-payment, even when willingness to pay exists. The actual scale, characteristics, and clinical outcomes of uninsured patients in Japan remain largely unknown.

Materials and methods

We conducted a retrospective chart review at a small suburban hospital in Japan. The characteristics of patients without any form of health insurance at the time of the encounter were extracted from medical records from October 2021 to September 2025. Patient consent was obtained on an opt-out basis.

Results

Over the four-year study period, 20 patients received medical care at the hospital without any form of health insurance. Twelve patients (60%) required hospital admission at their first encounter. Nine patients (45%) were diagnosed with psychological disorders. Severe functional impairment or death occurred in 10 patients (50%), including two cases of death. Prior to hospital presentation, four patients (20%) had attempted to apply for public assistance at a municipal office but were denied.

Conclusions

Some individuals who remained outside the national insurance system present with severe and preventable illnesses. Although this study cannot establish a causal relationship, lack of insurance coverage may contribute to delayed care and adverse health outcomes. Efforts to remove administrative barriers and strengthen social work support may help reduce preventable harm and promote health equity within Japan's healthcare system.

## Introduction

Universal health coverage is essential for protecting population health and ensuring the fundamental human right to access medical care [1]. Japan established a universal health insurance system in 1961, requiring all residents to enroll in either employment-based insurance or the National Health Insurance (NHI) [2]. This system has contributed greatly to social stability and improved population health by enabling equitable access to medical services.

Despite the formal framework of universal coverage, gaps remain. National data indicate that a proportion of residents are effectively uninsured due to unpaid premiums or administrative restrictions. As of 2024, among the 16,075,041 households enrolled in NHI, 1,829,802 (11.4%) had unpaid premiums, 337,142 households (2.1%) held short-term insurance certificates with limited validity, and 77,413 households (0.4%) had eligibility certificates requiring full up-front payment at the time of care [3]. Thus, approximately 2.5% of households enrolled in NHI experience effectively uninsured conditions. Individuals facing unstable employment or job loss are particularly vulnerable to premium non-payment, even when willingness to pay exists [4].

Lack of insurance or financial hardship is known to delay care-seeking and contribute to preventable morbidity [5-7]. Previous reports from the Japan Federation of Democratic Medical Institutions, a nationwide network of more than 1,700 healthcare facilities in Japan, have documented preventable deaths related to delayed medical care for financial reasons, reporting 48 cases in 2024 alone [8]. However, these reports rely on voluntary case submission and do not reflect the full picture. To our knowledge, systematic, hospital-based data on uninsured encounters in Japan is lacking. Thus, the actual scale, characteristics, and clinical outcomes of uninsured patients in Japan remain largely unknown.

This study aims to describe the number of uninsured encounters, patient characteristics, and clinical outcomes in a small community hospital over a four-year period.

## Materials and methods

Setting

This retrospective chart review was conducted at Ehime Seikyo Hospital, a small suburban hospital in Matsuyama, Japan, where the first and second authors were employed. The hospital has 88 beds and primarily functions as a community-based primary care facility, consistent with the role of small hospitals in Japan [9]. Clinical departments include family medicine, internal medicine, orthopedics, pediatrics, psychiatry, and surgery. The facility manages approximately 230 outpatient visits per day and admits around 1,700 inpatients annually. The hospital is committed to non-discriminatory, equitable care delivery, implements the Free/Low-Cost Medical Care Program [10], and has extensive experience in supporting patients with social and medical complexity [11].

Participants

Medical records from October 2021 to September 2025 were reviewed. Eligible participants were individuals who received medical care at the hospital without any form of health insurance at the time of the encounter. In this study, "no form of health insurance" was defined as the absence of valid enrollment in any public or employment-based health insurance scheme that would permit routine insured medical care at the time of presentation. This category included individuals who had lost insurance coverage because of administrative or financial circumstances and those who were not enrolled in any insurance scheme despite being legally required to do so. Patients who possessed valid insurance eligibility but did not present their insurance card during the visit were excluded. Individuals with active insurance coverage, including those covered by public assistance, employment-based insurance, or NHI, were not eligible.

The first author used the electronic medical record system to identify potentially eligible patients through registration and billing records indicating uninsured status at the time of presentation. Cases were identified using registration status information documenting the absence of insurance coverage and records of full self-payment at the initial encounter. Insurance status was subsequently verified through review of clinical notes, administrative records, and medical social work records. Patients who had valid insurance eligibility but failed to present documentation were excluded after verification.

Because the hospital had a standardized process in which all uninsured patients were automatically referred to a medical social worker, the second author, who served in this role, possessed detailed knowledge of each case. To reduce potential misclassification, the first and second authors independently reviewed all potentially eligible cases and compared their assessments. Disagreements regarding eligibility or classification were resolved through discussion and consensus. During data classification, a small number of discrepancies regarding diagnostic categorization and outcome classification were identified and resolved through consensus review. No independent third-party audit was performed.

Data collection and analysis

The following variables were extracted from the electronic medical records: age, sex recorded in the family register, reason for visit, diagnosis (main diseases causing initial visits or admissions and coinciding psychiatric disorders), details of treatment and social work involvement, presence of disabling sequelae, and insurance status at the final follow-up. Classification of diagnoses, disabling sequelae, and final insurance categories was independently assessed by two authors, and discrepancies were resolved through consensus. Disabling sequelae were defined as impairments that, based on the available clinical records, the authors considered sufficiently likely to persist after treatment and to have a meaningful impact on daily functioning. Data were collected at the patient level rather than at the individual visit level. Analyses were based on information obtained throughout the entire course of care, incorporating all relevant findings documented across multiple encounters.

Data were analyzed descriptively. Continuous variables are presented as medians with interquartile ranges (IQRs), and categorical variables are presented as counts and percentages. Percentages were calculated using the total number of study participants as the denominator unless otherwise specified and were rounded to the nearest whole number. All data were compiled and analyzed using Microsoft Excel (Version 2024, Microsoft Corporation, Redmond, Washington, United States).

No inferential statistical analyses, including confidence intervals or hypothesis testing, were performed. Given the small sample size and the descriptive objective of the study, inferential analyses were considered inappropriate. The purpose of this study was to describe the characteristics and clinical outcomes of uninsured patients encountered at a single institution rather than to test hypotheses or estimate population-level associations.

Data completeness was assessed during chart review by confirming the availability of all predefined study variables for each participant. Information was obtained from multiple sources within the electronic medical record, including clinical notes, administrative records, and social work documentation. No missing data were identified for the variables included in the analysis. Socioeconomic variables such as employment status, housing status, and immigration status were not consistently documented in the medical records and were therefore not included in the study.

Ethical consideration

Patient consent was obtained on an opt-out basis. A notice about the study was posted in the hospital waiting room and on the website. Patients who did not want their data to be used for the study were given the opportunity to refuse. To protect patient confidentiality, all data were extracted and analyzed in anonymized form. Direct personal identifiers were removed before analysis, and the dataset was stored on password-protected hospital computers accessible only to the research team. The study was approved by the Ethical Committee of Ehime Seikyo Hospital (approval number: 51-2-2025-006).

## Results

Over the four-year study period, 20 patients received medical care at the hospital without any form of health insurance. The median age was 55.3 years (IQR: 45.8-65.8). Sixteen participants (80%) were male, and four (20%) were female. Twelve patients (60%) required hospital admission at their first encounter (Table 1).

**Table 1:** Participants' demographics (n=20)

Demographics	Value
Age (years), median (IQR)	55.3 (45.8-65.8)
Male, n (%)	16 (80%)
Female, n (%)	4 (20%)
Inpatient, n (%)	12 (60%)
Main disease, n (%)	
Diabetes mellitus	4 (20%)
Cardiac diseases	3 (15%)
Acute exacerbation of chronic heart failure	2
Hypertension	1
Cerebral diseases	3 (15%)
Stroke	1
Epilepsy	1
Wernicke-Korsakoff encephalopathy	1
Psychiatric disorders	3 (15%)
Depression	2
Dementia	1
End-stage malignancies	2 (10%)
Lung cancer	1
Rectal cancer	1
Musculoskeletal diseases	2 (10%)
Fracture	1
Costochondritis	1

The primary conditions prompting medical care included diabetes mellitus (n=4; 20%), cardiac diseases (n=3; 15%), cerebral diseases (n=3; 15%), psychiatric disorders (n=3; 15%), end-stage malignancies (n=2; 10%), and musculoskeletal diseases (n=2; 10%) (Table 1). In total, nine patients (45%) were diagnosed with psychiatric disorders (both main diagnoses and concurrent diagnoses) during the study period (Table 2).

**Table 2:** Concurrent psychiatric disorders (n=9)

Disease	N
Depression	3
Dementia	2
Alcoholism	2
Intellectual disability	1
Anxiety disorder	1

Severe functional impairment or death occurred in 10 patients (50%). Conditions included death after discharge (n=2), motor dysfunction (n=2), neurocognitive dysfunction (n=2), cardiac dysfunction (n=2), and visual impairment (n=2) (Table 3).

**Table 3:** Aftereffects causing impaired dysfunction including death (n=10)

Dysfunction	Value
Death	2 (10%)
End-stage malignancy	2
Motor dysfunction	2 (10%)
Stroke	1
Ischemic lower limb	1
Neurocognitive dysfunction	2 (10%)
Wernicke-Korsakoff encephalopathy	1
Alcoholic encephalopathy	1
Cardiac dysfunction	2 (10%)
Heart failure after myocardial infarction	2
Visual impairment	2 (10%)
Diabetic retinopathy	2

Prior to hospital presentation, four patients (20%) had attempted to apply for public assistance at a municipal office but were denied. Medical social workers provided support for insurance acquisition in all cases. As a result, 10 patients (50%) successfully enrolled in public assistance insurance schemes. Eight patients obtained NHI, of whom three received additional support through the Free/Low-Cost Medical Care Program. One patient secured employee health insurance. One patient relocated and remained without insurance at the time of transfer.

## Discussion

This study demonstrated that 20 uninsured individuals sought medical care at a small primary care hospital over a four-year period and half experienced severe functional impairment or death. These findings suggest substantial inequities in timely access to care among uninsured individuals within Japan's health system. Although this study cannot establish a causal relationship between uninsured status and adverse outcomes, the severity observed at presentation raises concerns about barriers to timely access to healthcare among uninsured individuals.

Previous research has shown that financial barriers [12-14], social isolation [15,16], and stigma [17,18] can influence care-seeking behavior and healthcare utilization. In this study, many patients presented with chronic, progressive, or psychiatric conditions, and 45% were diagnosed with at least one psychiatric disorder during the study period. The observed psychiatric conditions included depression, dementia, alcoholism, intellectual disability, and anxiety disorder. These findings may suggest a substantial burden of mental health and cognitive conditions among uninsured patients.

Another notable finding was that 20% of participants attempted to apply for public assistance before presenting to the hospital but were denied at the municipal office. Previous studies and reports have described administrative practices that may discourage or impede access to welfare benefits among individuals who may be eligible for support [19]. Such barriers may contribute to difficulties in obtaining insurance coverage or financial protection and may further exacerbate health inequities. Although our data cannot determine the frequency or causes of these experiences, they suggest that administrative processes may represent an important obstacle for some uninsured individuals.

Medical social work played a pivotal role in connecting patients with insurance coverage and social support. In this study, all uninsured patients were referred to a medical social worker, who assisted with insurance enrollment procedures, applications for public assistance, coordination with municipal agencies, and linkage to available financial support programs, including the Free/Low-Cost Medical Care Program. Most patients subsequently obtained some form of insurance coverage. These findings highlight the potential value of integrating social care services into primary care settings, particularly for patients facing complex medical, financial, and administrative challenges [20,21].

Limitations

This study has several limitations. First, it was a retrospective chart review conducted at a single small community hospital, limiting the generalizability of the findings to other regions, urban settings, or healthcare institutions in Japan.

Second, the sample size was small (n=20). Because the study was intended to be descriptive, no inferential statistical analyses were performed; however, percentages derived from a small number of cases should be interpreted cautiously.

Third, selection bias is possible. The study hospital has a longstanding commitment to caring for socially marginalized populations, operates the Free/Low-Cost Medical Care Program, and maintains a well-established medical social work system. Hospitals without similar support structures may encounter different patient populations and outcomes. Conversely, uninsured individuals who never sought medical care were not captured by this study, potentially leading to the underestimation of the burden of unmet healthcare needs among uninsured populations.

Fourth, although participant identification involved a structured verification process by two investigators, some misclassification of insurance status remains possible because the study relied on retrospective chart review. In addition, the second author's involvement as both a medical social worker and reviewer may have introduced observer bias, although independent review and consensus procedures were used to reduce this risk.

Finally, socioeconomic variables such as employment status, housing status, immigration status, educational attainment, and social support were not consistently documented in the medical records and therefore could not be analyzed. As a result, the social mechanisms underlying uninsured status and adverse health outcomes could not be examined in detail.

## Conclusions

This study found that uninsured patients continue to seek care in Japan despite the existence of universal health coverage and many presented with severe or advanced illness. Although the present study cannot establish causal relationships, financial insecurity, administrative barriers, and limited access to timely healthcare may contribute to the challenges faced by uninsured individuals. Strengthening collaboration between healthcare providers and social support services may help improve access to care for socially marginalized populations. Efforts to reduce administrative barriers, improve insurance accessibility, and enhance social work support warrant further consideration as potential strategies to promote health equity within Japan's healthcare system.
